# Wear Management of Colored Foils for the Assessment of Sleep Bruxism Patterns—A Prospective, Randomized Crossover Study

**DOI:** 10.3390/diagnostics13020172

**Published:** 2023-01-04

**Authors:** Ferida Besirevic-Bulic, Martina Schmid-Schwap, Michael Kundi, Benedikt Sagl, Eva Piehslinger

**Affiliations:** 1 Department of Prosthodontics, University Clinic of Dentistry, Medical University of Vienna, Sensengasse 2a, 1090 Vienna, Austria; 2 Center for Public Health, Department of Environmental Health, Medical University of Vienna, Kinderspitalgasse 15, 1090 Vienna, Austria; 3 Center of Clinical Research, University Clinic of Dentistry, Medical University of Vienna, Sensengasse 2a, 1090 Vienna, Austria

**Keywords:** sleep bruxism, diagnostic bruxism foil, tooth grinding, abrasion of teeth

## Abstract

The assessment of bruxism relies on clinical examinations, questionnaires, and polysomnography. The additional use of colored foils (BruxChecker^®^) could enable a more precise evaluation of bruxing patterns. To assess differences between use of the foils during stress periods or just on consecutive nights and to determine a reasonable duration of using the foils, 28 patients were classified according to the Research Diagnostic Criteria for Temporomandibular Disorders (RDC/TMD) and were randomly assigned to wearing the 12 foils for six consecutive nights (alternately in the upper and lower jaw; “consecutive”) or six nights within one month following days of high stress (“stress”) in a crossover design. The sizes of the attrition areas were measured with ImageJ. Stress was evaluated using the Perceived Stress Scale. The Stress Coping Questionnaire (SVF-120) was used for assessing habitual stress coping strategies. Areas of attrition increased significantly from day 1/2 to 5/6, both for the upper and lower jaw. Molars in the mandible had significantly larger attrition areas than in the maxilla. No significant differences were detected between “consecutive” and “stress” phases. The foils were suitable for differentiating teeth more or less affected by bruxism and were able to show that areas of attrition increased with days, indicating that some adaptation takes place and several days of wearing the foils are required to show the full picture. However, no differences between low/moderate- and high-stress phases were detected.

## 1. Introduction

Sleep bruxism is defined as masticatory muscle activity during sleep that is characterized as rhythmic (phasic) or non-rhythmic (tonic). It was described that, in otherwise healthy individuals, bruxism should not be considered as a disorder, but rather as a behavior that can be a risk (and/or protective) factor for certain clinical consequences [1].

Symptoms and consequences of sleep bruxism include teeth attrition, hypermobility and hypersensitivity of teeth, hypertrophy and pain of masticatory muscles, headache, temporomandibular joint (TMJ) pain, fatigue of masticatory muscles, and temporomandibular disorders [2,3,4,5,6,7]. It may manifest as impressions of the rows of teeth on the tongue and cheek mucosa [8].

Epidemiological studies revealed a prevalence of 10–13% for sleep bruxism in adults [9] and 40–50% in children [10,11]. Historically, bruxism has been commonly associated with morphological factors (facial skeleton, dental occlusion), but more recent literature focusses on a combination of psychosocial and psychological factors such as depression, fear, or stress and exogenous factors as well as pathophysiological risk factors (e.g., smoking, alcohol, and drugs) [12,13,14,15]. The influence of psychological stress on sleep bruxism is a highly contested topic in dental literature. Stress has been reported to play a crucial role in the development and persistence of bruxism, as well as in its frequency and severity [16,17,18]. In contrast, no significant correlation was found between self-reported perceived stress according to the Perceived Stress Scale (PSS-10), depression, and bruxism [19]. It has been reported that having poor coping skills is a possible personality feature that has been associated with increased bruxism [20]. Neurological disorders and a genetic and/or familial predisposition may also be involved in the pathogenesis of bruxism [21]. It was reported that sleep bruxism is centrally mediated, with a complex interaction of all factors influencing the autonomic system during sleep. Awake bruxism was mainly associated with psychosocial factors [22,23].

Various methods (instrumental, non-instrumental procedures) to diagnose bruxism have been recommended [24]. A grading system for the operationalization of bruxism diagnosis has been introduced by an international consensus. It defines possible sleep bruxism as based solely on a positive self-report, probable sleep bruxism as based on a positive clinical inspection with or without a positive self-report, and definite sleep bruxism as based on a positive instrumental assessment with or without a positive self-report and/or a positive clinical inspection [1]. Non-instrumental procedures to assess and diagnose sleep and awake bruxism include self-reported questionnaires and/or clinical inspection [1]. The clinical examination is divided into an extraoral and an intraoral evaluation. The extraoral evaluation includes the jaw muscles, the TMJ, the presence of pain (e.g., teeth soreness and/or hypersensitivity, jaw muscle pain, TMJ pain, headache), and functional symptoms (e.g., difficulty in opening the mouth wide on awakening). The intraoral inspection encompasses complete dental examination (e.g., tooth wear, tooth enamel chippings, cracks and fractures of natural teeth, restorations failure, periodontal ligament thickening) and an inspection of the cheek and tongue mucosa (e.g., linea alba, tongue scalloping, traumatic lesions) [25]. The gold standard for instrumental methods to assess and diagnose sleep movement disorders and sleep bruxism is polysomnography (PSG) [26]. PSG involves various recordings including electromyography (EMG), electroencephalogram (EEG), electrocardiogram (ECG), and audiovisual recordings. PSG is an accurate but complex and expensive method requiring special equipment and has to be carried out in a sleep laboratory [27,28]. EMG devices have been used in recent years as a valid option for an easier approach to a definitive diagnosis of motoric activity [29,30,31].

The Research Diagnostic Criteria for Temporomandibular Disorders (RDC/TMD) has been the most commonly used diagnostic protocol for TMD research since its publication in 1992 [32]. This classification for TMD is internationally recognized and can be used to describe a clinical sample and for assessing potential relationships between bruxism patterns and TMJ health. The relationship between bruxism and TMD is controversial; however, the primary goal of the present study is not to assess this relationship but to determine whether the bruxism pattern or intensity is related to any clinical characteristic of TMJ health.

A less costly and easy-to-use alternative to evaluate sleep bruxism is the combination of a clinical examination and the home-use of colored dental pressure molding foils [33,34]. The BruxChecker^®^ (Scheu-Dental GmbH, Iserlohn, Germany) is a 0.1 mm thick polyvinyl chloride foil, coated on one side with red food colorant, developed by Sato and colleagues to evaluate grinding patterns as well as the individual occlusal schemes during sleep bruxism [33]. It has been confirmed that BruxChecker^®^ could be useful for the screening of sleep bruxism [35].

Overall, the aim of this prospective, randomized crossover study was to determine the optimal wear duration of the BruxChecker^®^ by measuring attritions of maxillary and mandibular surfaces. To assess the influence of perceived stress, two scenarios, wearing the foils on consecutive nights or nights after stressful days, were included in the study. The null hypothesis was that wear duration and wearing the foils on consecutive versus “stress” days has no influence on grinding patterns. Further aspects that should be addressed are the relationship of bruxism patterns to the RDC/TMD groups and the correlations between stress coping strategies and bruxism patterns.

## 2. Materials and Methods

This prospective, randomized crossover study was conducted at the special outpatient clinic for functional disorders at the University Clinic of Dentistry of the Medical University of Vienna. The patients included in this study were patients who after self-report and clinical inspection were classified as having probable sleep bruxism according to the classification system described above. Patients meeting all the following criteria were included: female and male adults ≥18 years of age, ability to open mouth more than 40 mm of incisal edge distance, closed row of teeth, full contact of all posterior teeth, clinical symptoms (e.g., grinding facets visible, tongue and/or cheek impressions, masseter and temporalis hypertrophy), increased muscle pain in the morning, extended jaw angle in the panoramic radiograph and at least two sites with tenderness in the closing masticatory muscles. The exclusion criteria were total or partial prostheses; alcohol abuse; use of soporifics, sedatives, or narcotics; epilepsy; pregnancy; and open, cross, or scissor bite of more than one tooth. The study was approved by the ethics committee of the Medical University of Vienna, Austria (No. 1682/2016). The practical part of the study was conducted from 2018 to 2019. Participation in the study was voluntary, and all participants provided written informed consent. The study was not blinded.

A total of 28 patients including 20 females (71%) and 8 males (29%) were included in the study.

Patients were classified according to the Research Diagnostic Criteria for Temporomandibular Disorders (RDC/TMD). This method was originally developed for scientific purposes and differentiates between axis I and II [32]. While axis I contains detailed criteria for the diagnosis of patients with temporomandibular disorders (TMDs) including myogenic dysfunction (group I), disc displacement (group II), and other joint diseases such as arthralgia and arthritis (group III); axis II deals with patients’ psychosocial status [36,37,38,39,40,41,42,43,44]. Axis II includes the chronic pain grade (CPG) score, a questionnaire for grading pain severity into four classes: Grade I, low disability–low intensity; Grade II, low disability–high intensity; Grade III, high disability–moderately limiting; and Grade IV, high disability–severely limiting. Although typically not required for the diagnosis of bruxism, MRIs were taken from most patients for the diagnosis of disc displacement, degenerative TMJ diseases, and arthritis. MRI was not requested in patients without clinical symptoms of TMD such as temporomandibular joint (TMJ) sounds.

To assess the psychosocial status of the patients, two additional questionnaires regarding stress, including the Stress Coping Questionnaire (SVF 120) by Janke et al. [45] and the Perceived Stress Scale (PSS-10), were administered to determine possible associations between bruxism and stress exposure and coping strategies. The PSS-10 is among the most widely used self-administered stress scales in social sciences [46]. The PSS-10 was evaluated at three time points, before (first appointment before application of BruxChecker^®^), during use of the BruxChecker^®^, and after the use of the BruxChecker^®^ for both study phases. The Stress Coping Questionnaire (SVF-120) is used for assessing habitual stress coping strategies. The SVF 120 includes 20 subscales with 6 items each. Strategies are classified in two subgroups, namely stress-reducing (subtests 1–10) or stress-increasing (subtests 13–18) strategies. Stress-reducing strategies are termed “Positive Strategies” and stress-increasing strategies as “Negative Strategies”. Subtests 11 “Need for Social Support”, 12 “Avoidance”, 19 “Aggression”, and 20 “Medication Use” are not included in the positive or negative strategies [45].

**Figure 1:**
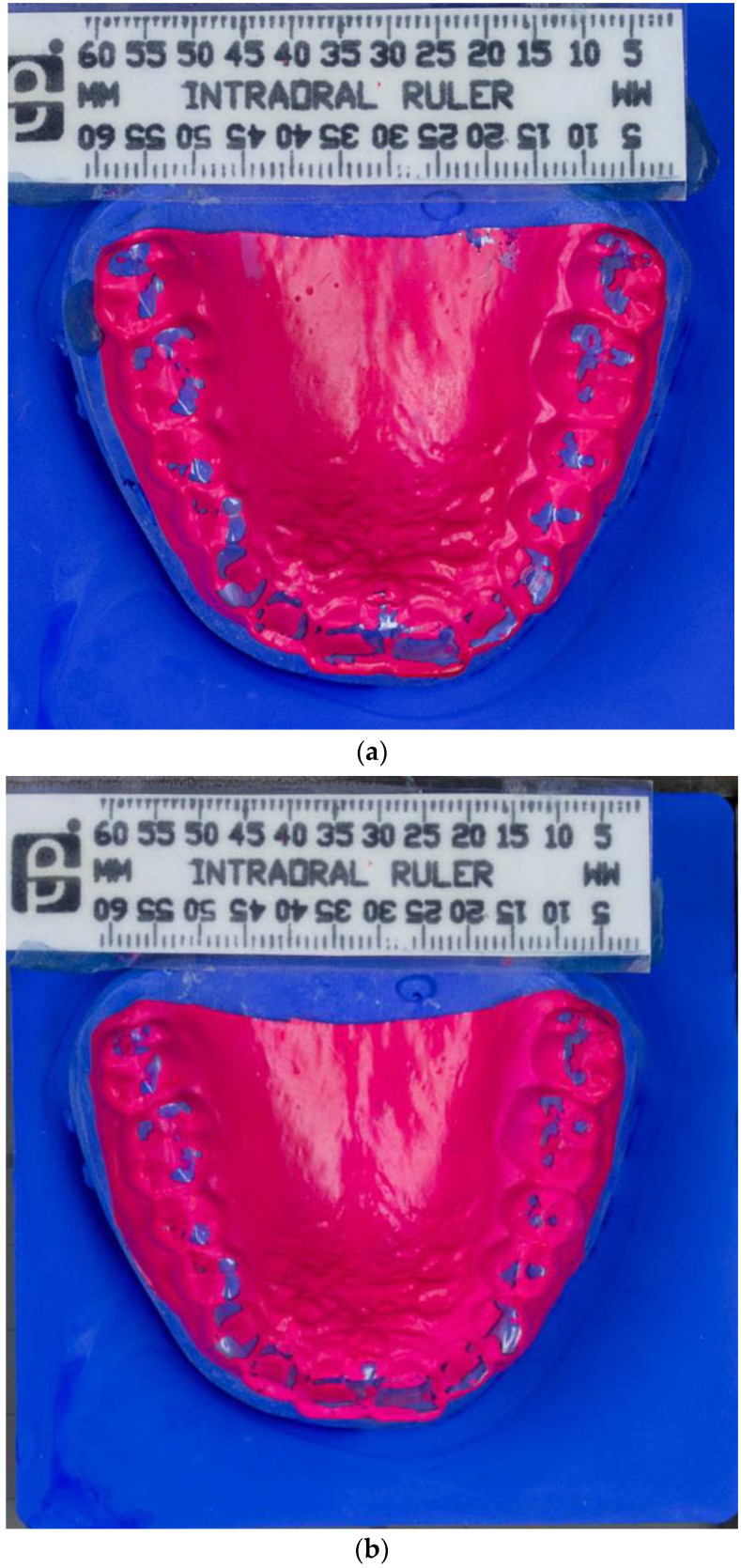
(**a**) Photograph of BruxChecker^®^ foil after wear “stress” phase. (**b**) Photograph of BruxChecker^®^ foil after wear “consecutive” phase.

Impressions of the upper and lower jaw were taken using alginate (Orthoprint Zhermack S.p.a., Badia Polesine, Italy), an irreversible, elastic, and hydro-colloidal impression material, for the preparation of the dental pressure molding foils. This foil was produced with heating to 230 °C using a vacuum deep-drawing device (Biostar, Scheu Dental, Iserlohn, Germany) and deep drawn for 15 s over dental plaster models from the upper and the lower jaw. The foil was trimmed in the vicinity of the marginal gingiva (Figure 1a,b).

After the BruxChecker^®^ was prepared by the dental laboratory of the University Clinic of Dentistry, patients received the devices in a hard box with six separate and numbered compartments for 6 colored foils (3 for the upper jaw and 3 for the lower jaw) in the two phases (consecutive, stress). Following the recommendations of the manufacturer, each foil was used only for one night for hygienic reasons. Patients were randomly assigned to either first wearing the BruxChecker^®^ for six consecutive nights without consideration of stress (“consecutive”) or for six nights following days subjectively assessed as high-stress days (“stress”) in a period of one month. Both phases were completed by all patients, with one group starting with the “consecutive” phase followed by the “stress” phase, while the other group completed the study phases in the reverse order. In both phases, foils were alternated between the upper and the lower jaw. Our decision to use 6 foils per phase was due to the ease of comparing the size of the attrition samples, because if the participant had only worn one foil for 6 nights, a huge linear and operational effort would be required to compare the sizes of the attrition area. Each patient in both phases had 1 month to wear the colored foils. In the consecutive phase, the patient decided within the month, which 6 consecutive nights to wear the foils. In the stress phase, the patient chose the nights following days with increased stress levels within the specified month. All patients selected the 6 nights after stressful days as demanded in the protocol.

**Figure 2:**
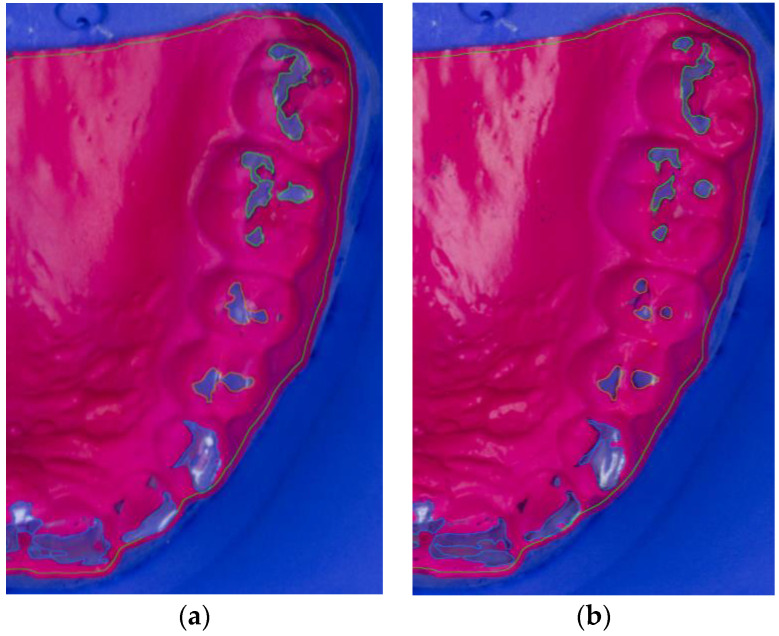
Segmented photograph (zoomed in) of BruxChecker^®^ foil after wear (**a**) “stress” phase; (**b**) “consecutive” phase.

After wearing the BruxChecker^®^, the foils were analyzed. First, they were placed on the plaster models made from blue gypsum (2/3 Sherasockel-Flüssiggips, SHERA Werkstoff-Technologie GmbH & Co. KG, Lemförde, Germany, and 1/3 hydro-stone^®^ 200, dentona AG, Dortmund, Germany) to assure good contrast with the red BruxChecker^®^ foils. Photographs for all foils were obtained using a standardized camera stand to keep the distance to the model constant. A ruler was used to relate image pixels to millimeters. The attrition areas were segmented into three tooth groups (front, premolar, molar) semi-automatically using the Fiji ImageJ package [47]. These segmented models were measured and statistically evaluated after the “stress” and “consecutive” phases (Figure 2a,b).

Area size as well as the location of the attrition were recorded for all patients. Grinding areas were normalized to the total surface area of the dental arch of the maxilla and the mandible separately, to account for differences in tooth size and to make the results more comparable between groups. Data were expressed as a fraction of the total area.

Sample size calculation was performed based on the assumption that an effect size corresponding to a partial eta-square (the proportion of the variance explained by the different sources of variation within the ANOVA model) of 0.04 (reflecting a small to medium effect size) should be detected with a power of 80% at a two-sided significance level of 5% assuming a correlation between attrition areas of 0.8. Under these assumptions, a sample size of *n* = 27 was required, which was increased to *n* = 28 to make the two groups with alternative sequences of “consecutive” and “stress” equal in size.

Data were analyzed with Stata 13.1 (StataSoft, College Station, TX, USA). Fractions of the attrition areas were square root transformed and compared by mixed model ANOVA with the within subject factors: “consecutive”/“stress”, mandible/maxilla, front/premolar/molar, and days (1/2, 3/4, and 5/6) and with the between subject factor sequence (“consecutive” → “stress” and “stress” → “consecutive”). Normality of residuals was tested by the Kolmogorov–Smirnov test with Lilliefors’ corrected *p*-values. Sphericity was determined by Mauchly’s tests and compound symmetry by Greenhouse–Geisser tests. Differences by RDC/TMD axis I and II subgroups were tested by the same procedure with sequences removed. The relationship between areas of attrition and the difference of attrition areas between “stress” and “consecutive” nights and coping strategies (SVF subscales) was analyzed by Spearman rank correlation. Analysis of the relationship between bruxism patterns and RDC/TMD groups as well as coping strategies must be considered exploratory, and no correction for multiple endpoints and multiple comparisons was applied.

## 3. Results

The mean age of the 20 females (71%) and 8 males (29%) was 31.2 ± 9.0 years (range, 22 to 55 years).

According to the RDC/TMD classification for axis I, a total of 21 patients (75%) were categorized into group I (myogenic dysfunction); 9 (32%) into group II (disc dislocation with repositioning of the right (*n* = 5), left (*n* = 2), and both joints (*n* = 2)); and 21 patients into group III including arthralgia in the right (*n* = 11) and/or left (*n* = 9) joint and/or arthritis (*n* = 10). Overall, 13 patients (46%) had moderate or severe unspecific physical symptoms with pain and 10 (36%) without pain (see also Table S1).

MRI assessment of the TMJ of 24 patients (86%) with potential pathologies after clinical examination revealed a disc displacement with (*n* = 12) or without (*n* = 4) reduction, and 5 patients showed incipient or high-grade degenerative disc changes or TMJ arthritis.

Concerning axis I, there was a statistically significantly higher difference between attrition areas after stress days as compared to consecutive nights for patients with disc displacement (group IIa 3.5% higher under stress vs. 0.3% higher in those without disc displacement). For axis II groups (unspecific physical symptoms exclusive of pain: moderate/increased), there was a significant difference regarding mean area after stress (*p* = 0.024) and consecutive nights (*p* = 0.043) with moderately increased symptoms showing the highest attrition areas. The difference between stress and consecutive nights in attrition area was statistically significant (*p* = 0.044) comparing those with Chronic Pain Scale group II and above with those below (4.1% vs. 0.1%) and comparing psychosocial groups (4.1% increased, 3.2% moderate psychosocial problem vs. no psychosocial problem −0.4%; *p* = 0.007; Table 1). Other details of assessment by RDC/TMD are shown in Supplementary Table S2.

BruxChecker^®^ foils showed a mean attrition area of 1% of the total area (95% confidence interval: 0.7–1.4%). Large differences (*p* < 0.001) were found between frontal (1.0%; 95% CI: 0.7–1.5%), premolar (0.6%; 95% CI: 0.4–0.9%), and molar teeth (1.7%; 95% CI: 1.2–2.5%) (Figure 2). A significant relative increase of attrition area from the first to the last night of 16% was observed (*p* = 0.042). No statistically significant difference between “stress” and “consecutive” nights occurred (Table 2). However, differences between “consecutive” and “stress” nights were significantly (*p* = 0.030) larger for the maxilla (13% larger attrition area on stress days) than for the mandible (7% smaller area on stress days).

**Figure 3:**
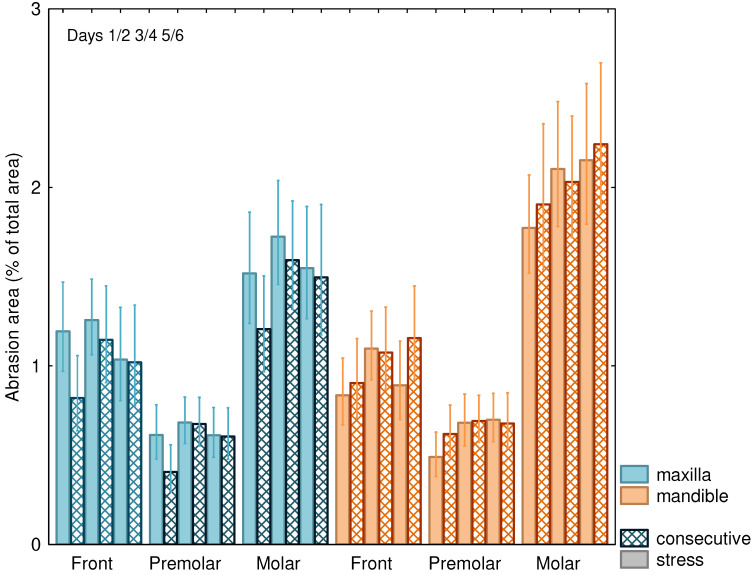
Attrition area (±SEM) in % of total area for “stress” nights and on consecutive nights for maxilla and mandible and front, premolar, and molar teeth.

The attrition area increased from night 1/2 to night 5/6 (*p* = 0.042; Table 2, Figure 3) especially for the mandible and was, in general, more pronounced on “consecutive” nights as compared to “stress” nights. The attrition areas of the mandibular molars were comparatively larger than those of the maxilla (Figure 3), while areas for the frontal and premolar teeth showed less difference between the mandible and the maxilla, resulting in a highly significant (*p* = 0.003) interaction effect (Table 2).

There was no significant correlation between Perceived Stress Scale scores and attrition areas and the difference in area between “stress” and “consecutive” nights (Table 3). Hence, irrespective of the phase of the experiment, stress during the day as measured by the PSS had no influence on the attrition area.

**Figure 4:**
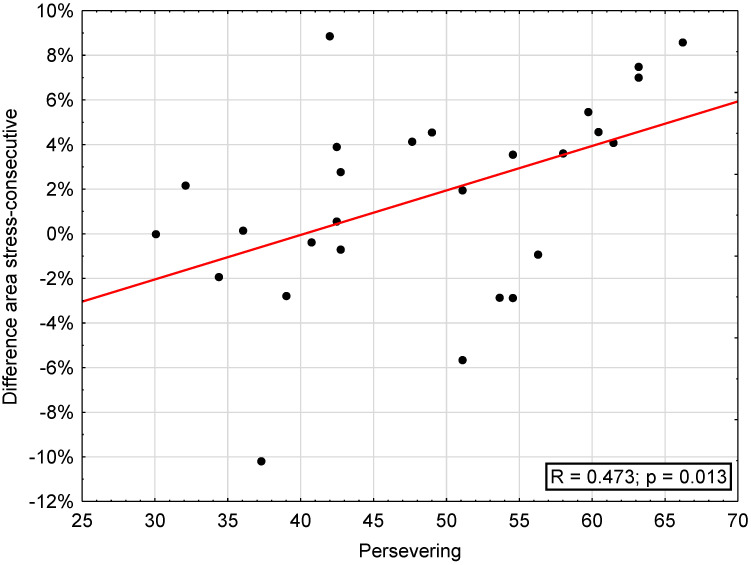
Correlation between difference of areas on “stress” and “consecutive” days and the subscale “persevering” of the coping questionnaire (SVF).

The correlation between attrition areas (“stress”, “consecutive”, difference “stress”–“consecutive”, maxilla, mandible) and SVF was significant regarding the difference stress-consecutive and trivialization (r = 0.392, *p* < 0.05) and reaction control (r = 0.421, *p* < 0.05), escape (r = 0.385, *p* < 0.05), perseveration (r = 0.473, *p* < 0.05; Figure 4), and self-blame (r = 0.419, *p* < 0.05) as well as regarding need for social support, showing a significant inverse correlation with area on consecutive days (r = −0.389, *p* < 0.05) and mandibular attrition area (r = −0.469, *p* < 0.05) (Table 4).

## 4. Discussion

We investigated the effect of wear duration and wear scenario of a colored thermoplastic foil for the visualization of attrition areas on the tooth surface caused by probable sleep bruxism. The colored foils are cheap, non-invasive, and easy to use [48,49]. Hokama et al. have shown that the occlusal foils are of value for the initial assessment of bruxism patterns [35]. While polysomnographic (PSG) examinations have been described as the gold standard for a definitive diagnosis of sleep bruxism [26], the method is expensive and the recording must be done in an unfamiliar environment [50]. However, as the best way to assess bruxism, Manfredini et al. described a combination of instrumental and non-instrumental approaches with special focus on the future design of multidimensional evaluation systems [51].

Accordingly, the use of colored foil could be considered a valuable, fast, and cost-effective first screening tool for bruxism. Most previous studies have used the BruxChecker^®^ for two nights with one foil per jaw [35,48,49,52]. Onodera et al. [33] reported that the BruxChecker^®^ was worn during sleep bruxism for two consecutive nights. Hokama et al. [35] described that the foil was worn for three consecutive nights but only the third night was evaluated. For diagnostic purposes, they assumed the first two nights to be the pre-assessment period to avoid bias [35]. Based on this research, we developed our investigation of the effect of wearing the colored foil on grinding area assessment. Our results showed that there was a significant increase of attrition area from the first to the last night. Consequently, our results support wearing the BruxChecker^®^ for a prolonged time to improve the quality of sleep bruxism assessment.

In addition to measuring the attrition areas, our study classified the patients by RDC/TMD. Moreover, patients with disc displacement showed statistically significantly larger attrition areas after stress days compared to consecutive nights. The relationship between sleep bruxism and TMDs is controversially discussed in dentistry [53]. The results of the published literature in the period 2009–2021 showed that the association between sleep bruxism and TMDs strongly depended on the assessment strategy for sleep bruxism [54].

To assess the psychosocial well-being of the patients and its possible connection with sleep bruxism, we used axis II of the RDC/TMD criteria (the Graded Chronic Pain Scale) [32,55], as well as PSS-10 and SVF-120 questionnaires in our study. Patients with high scores on the Chronic Pain Scale and impaired psychosocial well-being showed increased attrition areas in stress phases compared to consecutive phases. Our study found no connection between daily stress, assessed using PSS-10, and attrition area. This result is interesting since an association between stress and bruxism has been previously reported in several studies [49,56]. This relationship has been explained by stress being a hazard for the physiological or psychological integrity of an individual and may result in physiological and/or behavior-related responses [57]. Although these results seem to be in contradiction to our findings, other studies assessing the impact of stress on bruxism are between-subject studies, while our study is within subjects. It is still possible that the individual vulnerability to stress contributed to the development of bruxism in our patients, but the perceived daily stress does not influence tooth-grinding behavior.

As with any clinical study, some limitations to our approach remain. According to the ideas of a STAB (Standardized Tool for the Assessment of Bruxism) [58], bruxism diagnoses should be done using a multidimensional evaluation system. This approach integrates subject-based, clinically based, and instrumentally based assessments. We have focused on the first two and added a comprehensive investigation of grinding patterns. However, we did not perform a PSG that is one of the instrumentally based assessment tools. Secondly, our study did not use the current diagnostic criteria for temporomandibular disorders (DC/TMD), but used the RDC/TMD [59]. At the time of the ethical approval and the start of the study, the DC/TMD was not available in German and hence could not be used for the current study. While the use of the older diagnostic criteria is not ideal, TMD was not the focus of the presented study and the reported main limitations of the RDC lie in the detection of disc displacement without the use of MRI [60]. Since we collected MRI volumes for the majority of patients, the use of the RDC/TMD is a minor limitation. A further limitation of the study is that we did not include patients with suspected bruxism without subjective or clinical symptoms (e.g., pain, joint noises, or movement restriction).

Overall, the strengths of this study include the rigorous, randomized crossover approach and the precise measurement of the attrition areas. Furthermore, our results have clinically relevant implications concerning the ideal wear duration of the colored foils for bruxism assessment. Our results have shown that longer wearing of colored foil is useful for initial assessment of sleep bruxism.

## 5. Conclusions

In the present study, the BruxChecker^®^, a color-coated pressure molding foil, showed that areas of attrition increased with the number of nights of wear. There were no differences between consecutive nights and nights with high subjective stress, but this may be due to the comparatively high stress levels of the participants. Overall, our study suggests that wearing the foil on several consecutive nights alternating between the maxilla and the mandible may be sufficient to get a visual representation of sleep bruxism.

## Figures and Tables

**Table 1:** Attrition area (in percent of total area) under stress and on consecutive nights and difference between these areas (median and interquartile range) stratified for RDC/TMD axis I and II subgroups and MRI findings (*p*-values from ANOVA).

Group	Area “Stress”	Area “Consecutive”	Difference “Stress”–“Consecutive”
Total	25.6% (16.1%–35.9%)	26.0% (15.7%–35.9%)	1.2% (−1.2%–4.1%)
Axis I I no group	24.4% (17.6%–32.8%)	24.8% (12.4%–33.5%)	−0.4% (−1.3%–1.2%)
I a	27.3% (18.1%–37.4%)	27.2% (17.2%–39.2%)	3.2% (−1.4%–4.2%)
*p*	0.905	0.950	0.793
Axis I II no group	29.5% (15.9%–35.7%)	25.2% (16.7%–36.7%)	0.3% (−1.7%–3.8%)
II a	24.4% (22.1%–36.3%)	27.2% (13.7%–32.8%)	3.5% (−0.4%–7.5%)
*p*	0.966	0.720	0.047
Axis I III no group	24.4% (17.6%–32.8%)	24.8% (12.4%–33.5%)	−0.4% (−1.3%–1.2%)
III a	24.3% (18.7%–31.5%)	27.2% (17.8%–32.6%)	3.5% (−4.3%–5.7%)
III b	31.2% (18.7%–40.6%)	27.1% (17.5%–45.6%)	2.8% (0.0%–4.1%)
*p*	0.825	0.835	0.964
MRI no displ.	21.6% (16.4%–22.1%)	13.7% (13.5%–17.5%)	4.1% (2.8%–5.5%)
part.displ.	27.8% (21.4%–30.0%)	23.3% (19.5%–28.7%)	−0.9% (−1.4%–1.8%)
with red.	33.5% (20.9%–59.7%)	34.0% (18.5%–54.4%)	2.9% (−0.6%–4.2%)
w/o red.	33.3% (28.8%–34.8%)	32.8% (30.0%–32.8%)	0.5% (−1.2%–2.0%)
*p*	0.659	0.603	0.563
Axis II GCPS < G II	27.3% (22.3%–35.7%)	29.8% (19.7%–35.2%)	0.1% (−2.6%–3.2%)
G II+	21.6% (14.9%–40.6%)	17.5% (15.8%–37.0%)	4.1% (2.8%–4.5%)
*p*	0.843	0.624	0.044
Axis II depr. normal	27.8% (24.3%–35.4%)	32.4% (23.3%–35.6%)	-0.4% (−1.9%–0.5%)
moderate	22.5% (18.1%–45.4%)	20.3% (13.6%–41.4%)	3.2% (0.8%–4.3%)
increased	26.4% (16.2%–56.4%)	22.3% (16.7%–49.8%)	4.1% (2.6%–6.6%)
*p*	0.672	0.896	0.007
Axis II unsp.incl.pain normal	0.244 (0.183–0.322)	0.240 (0.176–0.328)	−0.014 (−0.049–0.001)
moderate	0.192 (0.300–0.230)	0.136 (0.312–0.191)	0.014 (0.037–0.035)
increased	0.132 (0.084–0.187)	0.165 (0.059–0.175)	−0.028 (−0.048–0.019)
*p*	0.289	0.357	0.449
Axis II unsp.excl.pain normal	27.8% (22.1%–35.7%)	32.4% (15.8%–37.0%)	0.5% (−0.9%–4.5%)
moderate	50.6% (33.0%–69.7%)	45.1% (29.4%–64.8%)	3.7% (1.9%–4.8%)
increased	15.9% (10.4%–20.9%)	17.0% (12.5%–20.5%)	1.0% (−2.1%–3.5%)
*p*	0.024	0.043	0.624
Axis II JDL none	24.4% (11.6%–38.2%)	24.8% (12.4%–36.3%)	0.1% (−0.5%–2.1%)
JDL ≥ 1	27.3% (20.9%–35.7%)	27.2% (18.7%–37.0%)	3.7% (−2.1%–4.5%)
*p*	0.950	0.954	0.585

Axis I Ia (myofascial pain), axis I IIa (disc displacement with replacement), axis I IIIa (arthralgia), axis I IIIb (osteoarthritis), MRI (magnetic resonance imaging) no displ. (without disc displacement), part. displ. (partial disc displacement), with red. (disc displacement with reduction), w/o red. (disc displacement without reduction), axis II GCPS (Graded Chronic Pain Scale), axis II depr. (depression scale score), axis II unsp.incl. pain (non-specific physical symptoms including pain), n. (normal), axis II unsp.excl.pain (non-specific physical symptoms excluding pain), axis II JDL(jaw disability list).

**Table 2:** Results of analysis of variance of attrition area. (Interaction indicated with *).

**Source of Variation**	**SQ**	**df**	**F**	***p***
stress/consecutive (S/C) upper/lower jaw (U/L) frontal/premolar/molar (FPM) days (T) S/C*U/L S/C*FPM U/L*FPM S/C*T U/L*T	0.061 0.461 34.692 1.501 0.514 0.002 1.146 0.156 0.106	1 1 2 2 1 2 2 2 2	0.228 1.543 19.715 3.361 5.272 0.055 6.638 0.310 0.776	0.637 0.225 <0.001 0.042 0.030 0.946 0.003 0.735 0.466
FPM*T S/C*U/L*FPM S/C*U/L*T S/C*FPM*T U/L*FPM*T S/C*U/L*FPM*T	0.120 0.032 0.404 0.086 0.026 0.105	4 2 2 4 4 4	1.092 0.778 1.636 1.164 0.336 0.886	0.364 0.464 0.204 0.331 0.853 0.475

SQ, sum of squares; df, degrees of freedom.

**Table 3:** Spearman correlations between Perceived Stress Scale (PSS-10) scores and attrition areas (all *p* > 0.05); PSS1: assessment before application of BruxChecker^®^, PSS2: during the wear phase of BruxChecker^®^, PSS3: at the end of the wear phase of BruxChecker^®^.

	Area Stress	Area Consecutive	Difference Area Stress–Consecutive	Area Maxilla	Area Mandible
PSS1 (before) PSS2 (during) PSS3 (after)	0.080 −0.085 −0.107	0.006 −0.144 −0.156	0.346 0.288 0.237	−0.010 −0.152 −0.151	0.104 −0.062 −0.100

PSS (1,2,3), Perceived Stress Scale (PSS-10).

**Table 4:** Spearman correlations between scores of the coping questionnaire (SVF) and attrition areas. Significant (*p* < 0.05) correlations indicated by (*).

	Area Stress	Area Consecutive	Difference Area Stress–Consecutive	Area Maxilla	Area Mandible
BAG	0.288	0.202	0.392 *	0.306	0.155
HER	−0.066	−0.115	0.237	−0.115	−0.056
SCHAB	0.100	0.075	0.116	0.103	0.064
ABL	−0.160	−0.149	−0.045	−0.147	−0.153
ERS	−0.107	−0.156	0.238	−0.123	−0.134
SEBEST	−0.126	−0.117	−0.036	−0.104	−0.134
ENTSP	0.150	0.116	0.154	0.205	0.038
SITKON	−0.051	−0.084	0.160	−0.084	−0.045
REKON	0.090	0.000	0.421 *	−0.001	0.096
POSI	−0.057	−0.132	0.359	−0.140	−0.035
BESOZU	−0.354	−0.389 *	0.185	−0.271	−0.469 *
VERM	−0.239	−0.305	0.326	−0.210	−0.329
FLU	0.173	0.090	0.385 *	0.154	0.095
SOZA	0.062	0.000	0.292	0.077	−0.027
GEDW	0.166	0.064	0.473 *	0.165	0.047
RES	0.136	0.067	0.320	0.180	0.000
SEMITL	0.310	0.238	0.322	0.323	0.197
SESCH	0.292	0.200	0.419 *	0.315	0.147
AGG	0.107	0.073	0.156	0.096	0.077
PHA	0.068	−0.001	0.324	0.041	0.021

BAG (trivialization), HER (de-emphasizing), SCHAB (denial of guilt), ABL (distraction, ERS (substitutive satisfaction), SEBEST (self-confirmation), ENTSP (relaxation), SITKON (situation control), REKON (reaction control), POSI (positive self-instruction), BESOZU (need for social support), VERM (avoidance), FLU (escape), SOZA (social isolation), GEDW (perseveration), RES (resignation), SEMITL (self-pity), SESCH (self-blame), AGG (aggression), PHA (medication use).

## Data Availability

Not applicable.

## Supplementary Materials

The following supporting information can be downloaded at https://www.mdpi.com/article/10.3390/diagnostics13020172/s1: Table S1. Distribution of patients into groups according to RDC/TMD and MRI. Table S2. Attrition area in percent of total area for maxillary and mandibular teeth (median and interquartile range) stratified for RDC/TMD axis I and II subgroups and MRI findings (*p*-values from ANOVA).

## References

[1] Lobbezoo F., Ahlberg J., Raphael K.G., Wetselaar P., Glaros A.G., Kato T., Santiago V., Winocur E., De Laat A., De Leeuw R. (2018). International consensus on the assessment of bruxism: Report of a work in progress. J. Oral Rehabil..

[2] Bader G., Lavigne G. (2000). Sleep bruxism; an overview of an oromandibular sleep movement disorder. Review article. Sleep Med. Rev..

[3] Kato S., Ekuni D., Kawakami S., Mude A.H., Morita M., Minagi S. (2018). Relationship between severity of periodontitis and masseter muscle activity during waking and sleeping hours. Arch. Oral Biol..

[4] Ahlberg J., Savolainen A., Rantala M., Lindholm H., Könönen M. (2004). Reported bruxism and biopsychosocial symptoms: A longitudinal study. Community Dent. Oral Epidemiol..

[5] McCutcheon N.B., Guile M.N. (1981). Stomach mucosal lesions in stressed rats with and without post-stress rest. Physiol. Behav..

[6] Serra-Negra J.M., Scarpelli A.C., Tirsa-Costa D., Guimarães F.H., Pordeus I.A., Paiva S.M. (2014). Sleep bruxism, awake bruxism and sleep quality among Brazilian dental students: A cross-sectional study. Braz. Dent. J..

[7] Strausz T., Ahlberg J., Lobbezoo F., Restrepo C.C., Hublin C., Ahlberg K., Könönen M. (2010). Awareness of tooth grinding and clenching from adolescence to young adulthood: A nine-year follow-up. J. Oral Rehabil..

[8] Pavone B.W. (1985). Bruxism and its effect on the natural teeth. J. Prosthet. Dent..

[9] Manfredini D., Winocur E., Guarda-Nardini L., Paesani D., Lobbezoo F. (2013). Epidemiology of bruxism in adults: A systematic review of the literature. J. Orofac. Pain.

[10] Manfredini D., Restrepo C., Diaz-Serrano K., Winocur E., Lobbezoo F. (2013). Prevalence of sleep bruxism in children: A systematic review of the literature. J. Oral Rehabil..

[11] Machado E., Dal-Fabbro C., Cunali P.A., Kaizer O.B. (2014). Prevalence of sleep bruxism in children: A systematic review. Dental Press J. Orthod..

[12] Lobbezoo F., Naeije M. (2001). Bruxism is mainly regulated centrally, not peripherally. J. Oral Rehabil..

[13] Manfredini D., Lobbezoo F. (2009). Role of psychosocial factors in the etiology of bruxism. J. Orofac. Pain.

[14] Manfredini D., Arreghini A., Lombardo L., Visentin A., Cerea S., Castroflorio T., Siciliani G. (2016). Assessment of anxiety and coping features in bruxers: A portable electromyographic and electrocardiographic study. J. Oral Facial Pain Headache.

[15] Manfredini D., Fabbri A., Peretta R., Guarda-Nardini L., Lobbezoo F. (2011). Influence of psychological symptoms on home-recorded sleep-time masticatory muscle activity in healthy subjects. J. Oral Rehabil..

[16] Arnold M. (1981). Bruxism and the occlusion. Dent. Clin. N. Am..

[17] Rugh J.D., Harlan J. (1988). Nocturnal bruxism and temporomandibular disorders. Adv. Neurol..

[18] Tsai C.M., Chou S.L., Gale E.N., McCall W.D. (2002). Human masticatory muscle activity and jaw position under experimental stress. J. Oral Rehabil..

[19] Smardz J., Martynowicz H., Michalek-Zrabkowska M., Wojakowska A., Mazur G., Winocur E., Wieckiewicz M. (2019). Sleep Bruxism and Occurrence of Temporomandibular Disorders-Related Pain: A Polysomnographic Study. Front. Neurol..

[20] Winocur E., Uziel N., Lisha T., Goldsmith C., Eli I. (2011). Self-reported bruxism–associations with perceived stress, motivation for control, dental anxiety and gagging. J. Oral Rehabil..

[21] Poveda Roda R., Bagan J.V., Díaz Fernández J.M., Hernández Bazán S., Jiménez Soriano Y. (2007). Review of temporomandibular joint pathology. Part I: Classification, epidemiology and risk factors. Med. Oral Patol. Oral Cir. Bucal.

[22] Lavigne G.J., Khoury S., Abe S., Yamaguchi T., Raphael K. (2008). Bruxism physiology and pathology: An overview for clinicians. J. Oral Rehabil..

[23] Huynh N., Kato T., Rompré P.H., Okura K., Saber M., Lanfranchi P.A., Montplaisir J.Y., Lavigne G.J. (2006). Sleep bruxism is associated to micro-arousals and an increase in cardiac sympathetic activity. J. Sleep Res..

[24] Lobbezoo F., Koyano K., Paesani D.A., Manfredini D. (2017). Sleep Bruxism. Principles and Practice of Sleep Medicine.

[25] Lobbezoo F., Jacobs R., De Laat A., Aarab G., Wetselaar P., Manfredini D. (2017). Chewing on bruxism. Diagnosis, imaging, epidemiology and aetiology. Ned. Tijdschr. Tandheelkd..

[26] Lavigne G.J., Rompré P.H., Montplaisir J.Y. (1996). Sleep bruxism: Validity of clinical research diagnostic criteria in a controlled polysomnographic study. J. Dent. Res..

[27] Lobbezoo F., van der Zaag J., van Selms M.K.A., Hamburger H.L., Naeije M. (2008). Principles for the management of bruxism. J. Oral Rehabil..

[28] Force P.T. (1997). American Sleep Disorders Association Standards of Practice Committee. Practice parameters for the indications for polysomnography and related procedures. Polysomnography Task Force. Sleep.

[29] Castroflorio T., Bargellini A., Rossini G., Cugliari G., Deregibus A., Manfredini D. (2015). Agreement between clinical and portable EMG/ECG diagnosis of sleep bruxism. J. Oral Rehabil..

[30] Manfredini D., Ahlberg J., Castroflorio T., Poggio C.E., Guarda-Nardini L., Lobbezoo F. (2014). Diagnostic accuracy of portable instrumental devices to measure sleep bruxism: A systematic literature review of polysomnographic studies. J. Oral Rehabil..

[31] Castroflorio T., Deregibus A., Bargellini A., Debernardi C., Manfredini D. (2014). Detection of sleep bruxism: Comparison between an electromyographic and electrocardiographic portable holter and polysomnography. J. Oral Rehabil..

[32] Dworkin S.F., LeResche L. (1992). Research diagnostic criteria for temporomandibular disorders: Review, criteria, examinations and specifications, critique. J. Craniomandib. Disord..

[33] Onodera K., Kawagoe T., Sasaguri K., Protacio-Quismundo C., Sato S. (2006). The use of a bruxchecker in the evaluation of different grinding patterns during sleep bruxism. Cranio.

[34] Tokiwa O., Park B.-K., Takezawa Y., Takahashi Y., Sasaguri K., Sato S. (2008). Relationship of tooth grinding pattern during sleep bruxism and dental status. Cranio.

[35] Hokama H., Masaki C., Mukaibo T., Tsuka S., Kondo Y., Hosokawa R. (2019). The effectiveness of an occlusal disclosure sheet to diagnose sleep bruxism: A pilot study. Cranio.

[36] Carlson C.R., Bertrand P.M., Ehrlich A.D., Maxwell A.W., Burton R.G. (2001). Physical self-regulation training for the management of temporomandibular disorders. J. Orofac. Pain.

[37] Epker J., Gatchel R.J. (2000). Prediction of treatment-seeking behavior in acute TMD patients: Practical application in clinical settings. J. Orofac. Pain.

[38] Garofalo J.P., Gatchel R.J., Wesley A.L., Ellis E. (1998). Predicting chronicity in acute temporomandibular joint disorders using the research diagnostic criteria. J. Am. Dent. Assoc..

[39] List T., Dworkin S.F. (1996). Comparing TMD diagnoses and clinical findings at Swedish and US TMD centers using research diagnostic criteria for temporomandibular disorders. J. Orofac. Pain.

[40] List T., Stenström B., Lundström I., Dworkin S.F. (1999). TMD in patients with primary Sjögren syndrome: A comparison with temporomandibular clinic cases and controls. J. Orofac. Pain.

[41] Marcusson A., List T., Paulin G., Dworkin S. (2001). Temporomandibular disorders in adults with repaired cleft lip and palate: A comparison with controls. Eur. J. Orthod.

[42] Phillips J.M., Gatchel R.J., Wesley A.L., Ellis E. (2001). Clinical implications of sex in acute temporomandibular disorders. J. Am. Dent. Assoc..

[43] Plesh O., Wolfe F., Lane N. (1996). The relationship between fibromyalgia and temporomandibular disorders: Prevalence and symptom severity. J. Rheumatol..

[44] Yap A.U.J., Chua E.K., Hoe J.K.E. (2002). Clinical TMD, pain-related disability and psychological status of TMD patients. J. Oral Rehabil..

[45] Beutel M.E., Brähler E. (2004). Testinformation. Diagnostica.

[46] Cohen S., Kamarck T., Mermelstein R. (1983). A global measure of perceived stress. J. Health Soc. Behav..

[47] Schneider C.A., Rasband W.S., Eliceiri K.W. (2012). NIH Image to ImageJ: 25 years of image analysis. Nat. Methods.

[48] Ommerborn M.A., Giraki M., Schneider C., Schaefer R., Gotter A., Franz M., Raab W.H.M. (2005). A new analyzing method for quantification of abrasion on the Bruxcore device for sleep bruxism diagnosis. J. Orofac. Pain.

[49] Tao J., Liu W., Wu J., Zhang X., Zhang Y. (2015). The study of grinding patterns and factors influencing the grinding areas during sleep bruxism. Arch. Oral Biol..

[50] Kato T. (2004). Sleep bruxism and its relation to obstructive sleep apnea-hypopnea syndrome. Sleep Biol. Rhythm..

[51] Manfredini D., Colonna A., Bracci A., Lobbezoo F. (2020). Bruxism: A summary of current knowledge on aetiology, assessment and management. Oral Surg..

[52] Park B.-K., Tokiwa O., Takezawa Y., Takahashi Y., Sasaguri K., Sato S. (2008). Relationship of tooth grinding pattern during sleep bruxism and temporomandibular joint status. Cranio.

[53] Manfredini D., Lobbezoo F. (2010). Relationship between bruxism and temporomandibular disorders: A systematic review of literature from 1998 to 2008. Oral Surg. Oral Med. Oral Pathol. Oral Radiol. Endod..

[54] Manfredini D., Lobbezoo F. (2021). Sleep bruxism and temporomandibular disorders: A scoping review of the literature. J. Dent..

[55] Von Korff M., Dworkin S.F., Le Resche L. (1990). Graded chronic pain status: An epidemiologic evaluation. Pain.

[56] Dao T.T., Lund J.P., Lavigne G.J. (1994). Comparison of pain and quality of life in bruxers and patients with myofascial pain of the masticatory muscles. J. Orofac. Pain.

[57] McEwen B.S., Wingfield J.C. (2003). The concept of allostasis in biology and biomedicine. Horm. Behav..

[58] Manfredini D., Ahlberg J., Aarab G., Bracci A., Durham J., Ettlin D., Gallo L.M., Koutris M., Wetselaar P., Svensson P. (2020). Towards a standardized tool for the assessment of bruxism (STAB)–Overview and general remarks of a multidimensional bruxism evaluation system. J. Oral Rehabil..

[59] Schiffman E., Ohrbach R., Truelove E., Look J., Anderson G., Goulet J.-P., List T., Svensson P., Gonzalez Y., Lobbezoo F. (2014). International RDC/TMD Consortium Network, International association for Dental Research; Orofacial Pain Special Interest Group, International Association for the Study of Pain Diagnostic Criteria for Temporomandibular Disorders (DC/TMD) for Clinical and Research Applications: Recommendations of the International RDC/TMD Consortium Network and Orofacial Pain Special Interest Group. J. Oral Facial Pain Headache.

[60] Galhardo A.P.M., da Costa Leite C., Gebrim E.M.M.S., Gomes R.L.E., Mukai M.K., Yamaguchi C.A., Bernardo W.M., Soares J.M., Baracat E.C., Gil C. (2013). The correlation of research diagnostic criteria for temporomandibular disorders and magnetic resonance imaging: A study of diagnostic accuracy. Oral Surg. Oral Med. Oral Pathol. Oral Radiol..

