# Supplementary material for: Wear Management of Colored Foils for the Assessment of Sleep Bruxism Patterns—A Prospective, Randomized Crossover Study
Source: Diagnostics (Basel). 2023 Jan 4;13(2):172. doi: 10.3390/diagnostics13020172 (PMC9858571; doi:10.3390/diagnostics13020172)
Supplement: Supplementary file 1 [file diagnostics-13-00172-s001.zip › diagnostics-2070252-supplementary.pdf]

Wear management of colored foils for the assessment of sleep bruxism patterns – A prospective randomized crossover study

Ferida Besirevic-Bulic [1], Martina Schmid-Schwap [1], Michael Kundi [2], Benedikt Sagl [3] and Eva Piehslinger [1]

[1] Department of Prosthodontics, University Clinic of Dentistry, Medical University of Vienna, Sensengasse 2a, 1090 Vienna, Austria
[2] Department of Environmental Health, Center for Public Health, Medical University of Vienna, Kinderspitalgasse 15, 1090 Vienna, Austria
[3] Center of Clinical Research, University Clinic of Dentistry, Medical University of Vienna, Sensengasse 2a, 1090 Vienna, Austria
* Correspondence: ferida.besirevic-bulic@meduniwien.ac.at

Relationship between attrition area of maxilla and mandible and RDC/TMD

The number of patients classified into the different groups according to RDC/TMD are shown in Table S1.

Concerning the relationship between diagnostic groups and attrition areas on maxillary and mandibular teeth, there was only a significant difference between Axis II groups with unspecific physical symptoms exclusive of pain regarding mean area of maxillary teeth attrition (p=0.026) and mandibular teeth (p=0.045) with the moderately increased symptoms group showing the highest area (Table S2).

Table S1. Distribution of patients into groups according to RDC/TMD and by MRI

Group		n	%
AXIS I I no group		7	25.0%
	I a	21	75.0%
AXIS I II right no group		21	75.0%
	II a	7	25.0%
AXIS I II left no group		24	85.7%
	II a	4	14.3%
AXIS I II left or right no group		19	67.9%
	II a	9	32.1%
AXIS I III right no group		7	25.0%
	III a	11	39.3%
	III b	10	35.7%

Group	n	%
AXIS I III left no group	9	32.1%
III a	9	32.1%
III b	10	35.7%
AXIS I III left or right no group	7	25.0%
III a	7	25.0%
III b	14	50.0%
MRI right no displ.	9	37.5%
part.displ.	4	16.7%
with red.	9	37.5%
w/o red.	2	8.3%
MRI left no displ.	8	33.3%
part.displ.	3	12.5%
with red.	11	45.8%
w/o red.	2	8.3%
MRI left or right no displ.	5	20.8%
part.displ.	3	12.5%
with red.	12	50.0%
w/o red.	4	16.7%
AXIS II GCPS<G II	19	67.9%
G II+	9	32.1%
AXIS II Depr normal	13	46.4%
moderate	9	32.1%
Increased	6	21.4%
AXIS II unsp.incl.pain n.	15	53.6%
moderate	8	28.6%
increased	5	17.9%
AXIS II unsp.excl.pain n.	18	64.3%
moderate	4	14.3%
increased	6	21.4%
AXIS II JDL none	11	39.3%
JDL≥1	17	60.7%

Axis I Ia (myofascial pain), Axis I IIa (disc displacement with replacement), Axis I IIIa (arthralgia), Axis I IIIb (osteoarthritis), MRI (magnetic resonance imaging) no displ (without disc displacement), part. displ. (partial disc displacement), with red. (disc displacement with reduction), w/o red (disc displacement without reduction), Axis II GCPS (Graded Chronic Pain Scale), Axis II depr. (depression scale score), Axis II unsp.incl. pain (non-specific physical symptoms included pain), n. (normal), AXIS II unsp.excl.pain (non-specific physical symptoms excluded pain), Axis II JDL(jaw disability list)

Table S2. Attrition area in percent of total area for maxillary and mandibular teeth (median and interquartile range) stratified for RDC/TMD Axis I and II subgroups and MRI findings (p-values from ANOVA).

Group	Area Maxilla	Area Mandible
Total	11.3% (8.0%-19.3%)	15.2% (7.8%-17.7%)
AXIS I I no group	10.9% (8.8%-17.1%)	13.7% (6.8%-16.0%)
I a	12.2% (8.3%-19.4%)	15.2% (9.4%-18.6%)
p	0.861	0.685
AXIS I II no group	11.0% (8.0%-18.9%)	15.4% (7.3%-17.8%)
II a	12.7% (9.8%-19.4%)	13.7% (9.5%-15.5%)
p	0.628	0.879
AXIS I III no group	10.9% (8.8%-17.1%)	13.7% (6.8%-16.0%)
III a	12.7% (8.4%-16.7%)	13.1% (8.6%-15.4%)
III b	11.7% (8.8%-19.4%)	17.5% (9.5%-21.3%)
p	0.915	0.654
MRT no displ.	9.8% (5.6%-10.2%)	9.4% (8.0%-9.5%)
part.displ.	10.4% (9.6%-14.0%)	15.2% (10.9%-15.4%)
with red.	16.0% (9.4%-23.6%)	17.6% (9.9%-31.5%)
w/o red.	16.6% (14.6%-18.0%)	15.2% (14.2%-15.8%)
p	0.811	0.435
AXIS II GCPS<G II	13.4% (9.8%-18.9%)	15.2% (11.7%-17.4%)
G II+	10.2% (7.0%-19.4%)	9.5% (6.6%-17.7%)
p	0.751	0.724
AXIS II Depr normal	14.1% (10.4%-17.9%)	15.5% (13.1%-17.7%)
moderate	9.8% (7.2%-25.4%)	13.2% (9.1%-18.0%)
increased	10.9% (7.8%-26.0%)	13.4% (6.8%-27.1%)
p	0.623	0.922
AXIS II unsp.incl.pain n.	16.6% (10.6%-19.3%)	15.6% (13.4%-19.5%)
moderate	9.9% (7.7%-31.5%)	11.2% (8.7%-20.7%)
increased	7.7% (7.0%-10.2%)	9.4% (6.0%-15.2%)
p	0.257	0.377
AXIS II unsp.excl.pain n.	14.1% (9.8%-19.3%)	15.5% (9.5%-17.8%)
moderate	25.1% (17.0%-34.9%)	22.8% (14.2%-32.4%)
increased	7.4% (5.4%-9.6%)	7.7% (4.9%-13.8%)
p	0.026	0.045
AXIS II JDL none	10.9% (7.4%-19.5%)	13.7% (5.8%-17.7%)
JDL≥1	12.2% (9.6%-19.3%)	15.2% (10.8%-18.4%)
p	0.786	0.740

Axis I Ia (myofascial pain), Axis I IIa (disc displacement with replacement), Axis I IIIa (arthralgia), Axis I IIIb (osteoarthritis), MRI (magnetic resonance imaging) no displ (without disc displacement), part. displ. (partial disc displacement), with red. (disc displacement with reduction), w/o red (disc displacement without reduction), Axis II GCPS (Graded Chronic Pain Scale), Axis II depr. (depression scale score), Axis II unsp.incl. pain (non-specific physical symptoms included pain), n. (normal), AXIS II unsp.excl.pain (non-specific physical symptoms excluded pain), Axis II JDL(jaw disability list)
